# 79602 Designing and Implementing an Assessment of Collaboration for a Clinical and Translational Research Community Advisory Board

**DOI:** 10.1017/cts.2021.611

**Published:** 2021-03-30

**Authors:** Paul Estabrooks, Keyonna King, Dave Palm, Heidi Keeler

**Affiliations:** University of Nebraska Medical Center

## Abstract

ABSTRACT IMPACT: This abstract presents a generalizable process to evaluate, and act on, community advisory board perceptions of collaboration effectiveness to improve clinical and translational research network function. OBJECTIVES/GOALS: Community advisory boards (CAB) play an important role in facilitating relevant and externally valid, clinical and translational research (CTR). The objective of this presentation is to describe a participatory process to derive collaboration metrics that can be used to assess CAB effectiveness. METHODS/STUDY POPULATION: During the 4th and 5th years of the Great Plains IDeA CTR award, we used a mixed-methods approach that included CAB (1) discussions related to the need to assess collaboration effectiveness, (2) review of the validated Wilder Collaboration Inventory to identify factors that, if maximized, would improve a sense of team science and enhance productivity, (3) planning for assessment frequency and follow-up processes, and (4) review of collaboration data to determine necessary actions for improvement. Qualitative data were gathered across components of the mixed-methods approach. Quantitative data were collected and reviewed by the CAB (n=11 members) during the 1st quarter of award year 5. RESULTS/ANTICIPATED RESULTS: CAB members expressed an interest in assessing collaboration effectiveness, identified important factors to assess, and agreed that annual assessment and follow-up would be appropriate. Key factors identified and assessed (5-point agreement scale--higher score reflects stronger agreement) were 1) mutual respect/trust (m=4.2); 2) appropriate cross section of members (m=3.6); 3) a shared stake in the process and outcomes (m=3.9); 4) flexibility in decision-making/collaboration (m=4.1); 5) roles and policy guidelines (m=3.6); 6) open communication (m=3.9); 7) goal achievement (m=4.0); 8) shared vision (m=4.0); and 9) skilled leadership (m=4.4). DISCUSSION/SIGNIFICANCE OF FINDINGS: CAB reflection on the initial collaboration assessment resulted in developing plans to broaden membership and clarify roles and policy guidelines related to CAB participation. There was strong consensus related to the utility of this assessment approach.

